# Risk of New-Onset Diabetes Mellitus as a Post-COVID-19 Condition and Possible Mechanisms: A Scoping Review

**DOI:** 10.3390/jcm12031159

**Published:** 2023-02-01

**Authors:** Prabal Chourasia, Lokesh Goyal, Dhruv Kansal, Sasmit Roy, Rohit Singh, Indrajeet Mahata, Abu Baker Sheikh, Rahul Shekhar

**Affiliations:** 1 Department of Hospital Medicine, Mary Washington Hospital, Fredericksburg, VA 22401, USA; 2 Department of Hospital Medicine, Christus Spohn Hospital Corpus Christ, Shoreline, TX 78404, USA; 3 Yale Waterbury Internal Medicine Program, Waterbury Hospital, Waterbury, CT 06708, USA; 4 Department of Nephrology, Centra Lynchburg General Hospital, Lynchburg, VA 24501, USA; 5 Department of Hemato-Oncology, University of Vermont Medical Center, Burlington, VT 05401, USA; 6 Department of Cardiology, University of Missouri, Springfield, MO 65804, USA; 7 Department of Internal Medicine, University of New Mexico Health Sciences Center, Albuquerque, NM 87106, USA

**Keywords:** new-onset diabetes mellitus, COVID-19 infection, post-COVID condition, long COVID-19

## Abstract

Long-term effects of COVID-19 are becoming more apparent even as the severity of acute infection is decreasing due to vaccinations and treatment. In this scoping review, we explored the current literature for the relationship between COVID-19 infection and new-onset diabetes mellitus four weeks after acute infection. We systematically searched the peer-reviewed literature published in English between 1 January 2020 and 31 August 2022 to study the risk of new-onset diabetes mellitus post-COVID-19 infection. This scoping review yielded 11 articles based on our inclusion and exclusion criteria. Except for one, all studies suggested an increased risk of new-onset diabetes mellitus 4 weeks after acute infection. This risk appears most in the first six months after the acute COVID-19 infection and seems to increase in a graded fashion based on the severity of the initial COVID-19 infection. Our review suggests a possible association of new-onset diabetes mellitus 4 weeks after acute COVID-19 infection. Since the severity of COVID-19 infection is associated with the development of post-infectious diabetes, vaccination that reduces the severity of acute COVID-19 infection might help to reduce the risk of post-COVID-19 diabetes mellitus. More studies are needed to better understand and quantify the association of post-COVID-19 conditions with diabetes and the role of vaccination in influencing it.

## 1. Introduction

COVID-19 infection, since it was first reported in December 2019 in Wuhan, China, has caused a global pandemic with significant morbidity and mortality affecting millions of people worldwide [1]. Concerted efforts across the globe have helped develop effective vaccines and treatments for COVID-19 [2]. While these have helped reduce the severity of acute infection, the long-term effects of COVID-19 have not been completely understood. The Centers for Disease Control and Prevention (CDC) has defined post-COVID-19 conditions as lingering or persistent symptoms of COVID-19 or the development of new health conditions not otherwise explained by other reasons four weeks after acute COVID-19 infection [3]. Per the CDC, up to 13.3% of patients at one month and more than 30% of hospitalized patients at six months can experience post-COVID-19 conditions [3].

Post-COVID conditions can be associated with a wide range of symptoms or conditions. As per the CDC, the most common symptoms of the post-COVID-19 condition include chest pain, difficulty concentrating, headache, sleep problems, dizziness, tiredness, shortness of breath, cough, change in smell or taste, diarrhea, stomach pain, and joint or muscle pain [3]. Though patients with severe COVID-19 are more at risk of post-COVID-19 conditions, it can be seen in both symptomatic and asymptomatic patients [3]. The patient population with an increased risk of developing post-COVID-19 conditions include patients needing hospitalization, patients with prior underlying health conditions before COVID-19 infection, unvaccinated persons, and people with multisystem inflammatory syndrome during or after acute COVID-19 infection [3,4]. Other risk factors for post-COVID-19 conditions include advanced age, body mass index, and female sex [5].

Patients with diabetes mellitus appear to have worse outcomes from acute COVID-19 infection [6]. Moreover, some studies have suggested that patients with COVID-19 may be at increased risk of developing new-onset diabetes mellitus in the post-infectious state, e.g., 4 weeks after acute infection [6]. The current literature suggests direct cytotoxic effects of the virus and inflammation among possible pathophysiology mechanisms [7,8]. In this scoping review, we explored the current literature for the relationship between COVID-19 infection and new-onset diabetes mellitus four weeks after acute infection. 

## 2. Materials and Methods

We systematically searched for peer-reviewed published studies in the English literature between 1 January 2020 and 31 August 2022 for new-onset diabetes mellitus in post-infectious COVID-19 patients. We conducted current scoping review in accordance with the PRISMA-ScR Checklist for the reporting of the results (Supplementary Table S1).

### 2.1. Study Definitions

We defined newly diagnosed diabetes mellitus as a new diagnosis of Hemoglobin A1C ≥6.5% or fasting blood glucose level ≥126 milligrams/deciliter (mg/dL) or two-hour blood glucose level ≥200 mg/dL with oral glucose tolerance test [9]. We also considered relevant ICD codes for diabetes mellitus in the reviewed studies where more information about new diabetes mellitus was unavailable. We defined post-COVID-19 conditions as new, returning, or ongoing health problems occurring ≥4 weeks after being infected with COVID-19 [3]. 

Inclusion criteria:

1. Development of diabetes mellitus at least four weeks after the initial COVID-19 infection.
2. Age ≥ 18 years.

Exclusion criteria:

1. Patients with a pre-existing diagnosis of diabetes or diagnosis of diabetes earlier than four weeks after the initial COVID-19 infection.
2. Exclusion criteria were kept to a minimum to review more relevant articles.

### 2.2. Data Sources and Search Strategy 

We utilized a three-step search strategy to find published studies in English literature. We performed an initial limited MEDLINE search to identify initial articles between 1 January 2020 and 31 August 2022. To develop a complete search strategy, we used text words in the tile, abstracts, and index terms to describe these articles.

We used the following combination of keywords with suitable Boolean operators: (COVID-19 OR “COVID 19” (MeSH Terms) OR COVID-2019 OR SARS-CoV-2 OR 2019-nCoV OR 2019-SARS-CoV-2) AND (survivor* OR recover* OR persistent OR follow up OR discharge* OR sequela* OR “post-acute” OR long COVID OR (“long term” AND follow up) OR 1 year OR one year OR 12 months OR twelve months”) AND (“diabetes mellitus, type 2”(mh) OR (diabetes*(tiab) AND (“noninsulin dependent”(tiab) OR type 2) to identify synonyms. Please refer to detailed search strategies for primary literature in Supplementary Table S2.

The databases searched included “MEDLINE”, “EMBASE”, and “SCOPUS”. We considered observational studies (cohort, case–control, cross-sectional, retrospective, prospective) for the proposed scoping review. We also cross-referenced the included studies for any additional studies that could be included. However, we excluded commentaries, conference proceedings, book chapters, and editorials. Evidence from qualitative studies was also not included in the review.

### 2.3. Data Extraction

Two independent reviewers (PKC and LG) screened all titles and abstracts based on inclusion and exclusion criteria. References with attached abstracts were retrieved from the databases above and uploaded into the excel sheet. Any disagreement between reviewers was resolved by a third reviewer (RS). We extracted data about the exposure of interest with exposure categories, populations, study methods, and outcomes based on the review question and specific objectives. We also recorded reasons for the exclusions of full-text studies not meeting the inclusion criteria (Supplementary Figure S1: PRISMA Diagram). 

### 2.4. Data Analysis

Data from included articles were extracted and summarized in an excel sheet and presented in tables.

## 3. Results

### 3.1. Literature Search Results

We identified 577 articles on our initial search with 62 duplicates that were removed before the screening. After screening, a total of 11 articles were found to be suitable for review.

### 3.2. Study Characteristics

Table 1 summarizes the key characteristics of the included studies. All the studies were conducted between 2020 and 2022. Out of the eleven studies, four were conducted in the USA, two in England and Italy, and one each in Spain, Germany, and China. Nine were cohort studies, including four prospective and five retrospective cohort studies, and there was one cross-sectional and one longitudinal prospective study. Five studies defined newly diagnosed diabetes with ICD codes [10,11,12,13,14]. Three studies described new-onset diabetes mellitus as per the American Diabetes Association (ADA) but did not use ICD codes [15,16,17]. Most studies discussed the incidence of type 2 diabetes, with a minority describing type 1 diabetes. The mean age was 60–65 years in five studies [10,16,17,18,19], whereas in others it ranged from 35 to 45 years [12,13,15]. Data regarding sex distribution were not available for two studies [11,13]. Except for two studies [13,14], females made up less than 50% of the population in the studies. The longest median follow-up was up to 365 days in three studies [15,18,19] and the shortest follow-up was 60 days [16]. Two studies noted steroid use during acute COVID-19 infection [13,17]. These studies had, on average, long-term follow-ups ranging from 2 months to 1 year after an acute COVID-19 infection.

The population included both outpatients and inpatients, including those who needed to be admitted to the intensive care unit. Except for four studies [14,15,16,17], the sample sizes in the studies were greater than 35,000 (35,865 to 600,055) and were statistically and clinically significant, improving the clinical significance of the results.

The result was reported as an incidence rate ratio (IRR) in six studies [8,11,12,13,16,18] (Table 2 and Table 3). Other studies described the odds ratio (OR) [11,19] and Hazard ratio [10,12] to describe the incidence of diabetes mellitus. Five studies [16,17,18,19,20] described outcomes in post-discharge (hospitalized) patients. Three studies described hospitalized patients [10,12,13], and three studies focused on outpatient COVID patients [11,12,13].

## 4. Discussion

With the emergence of new variants, the number of people with a history of COVID-19 infection continues to rise globally, thereby increasing the risk of post-COVID-19 conditions [21]. Even though effective vaccinations and treatments have made acute infections less severe, post-COVID-19 conditions can be seen in both patients with and without symptoms. One important sequela with significant implications for global disease burden is the increased risk of new-onset diabetes mellitus after COVID-19 infection. Multiple studies have shown that diabetes mellitus is a risk factor for severe COVID-19 infection and long COVID [6]. There is limited evidence of new-onset diabetes after acute COVID-19 infection [4,5,22,23,24,25,26]. In this scoping review, we explored the current literature for the relationship between COVID-19 infection and new-onset diabetes mellitus four weeks after acute infection.

The majority of studies had more males than females. One study found that the excess risk of new clinical sequelae did not differ between males and females [12]. In contrast, another study noted an association between new-onset diabetes and COVID-19 is more evident in males [19]. Multiple studies showed an increased risk of new-onset diabetes mellitus four weeks after acute COVID-19 infection [10,11,12,14,16,19,20]. The risk of new-onset diabetes mellitus appears to be highest in the first six months [11,12]. The risk of new-onset diabetes seems to increase in a graded fashion based on the severity of the acute infection [10,13], with critical illness being associated with a higher risk [19]. Subgroup analysis performed by Xie et al. noted a higher risk and burden of new-onset diabetes mellitus 4 weeks after acute COVID-19 infection in patients older than 65 years and black patients (compared with white patients), and in patients with prior cardiovascular disease, hypertension, hyperlipidemia, prediabetes, and higher body mass index [10]. A prior review by Joshi et al. discussed COVID-19-induced diabetes, primarily involving case series and a retrospective analysis in the pediatric population [27]. Sathish et al. included eight retrospective cohort studies with 3711 cases of COVID-19 and 492 cases of newly diagnosed diabetes [28]. 

Three studies [10,11,12] used viral infection as a comparator group, e.g., viral upper respiratory tract infections and influenza. They found an increased estimated risk or incidence of diabetes mellitus when compared to the viral upper respiratory tract and influenza infection. In the study by Rathmann et al., the incidence rate ratio was not increased for other forms of diabetes apart from type 2 diabetes [14]. A prior systematic review and meta-analysis by Shrestha et al., which included studies up to November 2020, explored the bi-directional relationship between COVID-19 and diabetes mellitus, including in patients with acute COVID-19 infection [29]. In comparison, our scoping review explored the risk of new-onset diabetes 4 weeks after acute COVID-19 infection, included studies up to August 2022, and included studies with acute upper respiratory tract infection and influenza patients as a control group. These additional included studies suggest that diabetes risk in the post-acute COVID-19 infection setting is higher when compared to other common viral infections.

Birabaharan et al. performed a subgroup analysis after excluding steroid use. They found that the risk of new-onset type 2 diabetes was lower in patients with mild COVID-19 but remained the same in patients with moderate to severe COVID-19 [11]. This study also noted that steroid use is associated with a higher risk of type 2 diabetes mellitus in mild COVID-19.

In contrast, a study by Laurenzi et al. noted an increase in fingerstick glucose during hospitalization. This study did not show an association between new-onset diabetes mellitus after the resolution of the acute infection, but this study was conducted with a relatively smaller sample size (*n* = 660) in a single institution [17]. In a study by Rezel-Potts et al., the risk of diabetes mellitus net incidence was elevated from weeks 5–12 but not from weeks 13–52 [15].

Vaccination may play a role in influencing the risk of new-onset diabetes in the post-acute COVID state by reducing the severity of acute illness. Four studies included populations during the time period when vaccination was available but did not account for the role of vaccination with regard to the risk of diabetes mellitus [10,11,15,19]. Xie et al. included a large cohort until 30 September 2021, including a population 10 months after vaccination was made available in the USA [30]. Seven studies that suggested an increased risk of diabetes included sicker patients needing hospitalization [10,12,13,16,18,19,20]. In two studies, the risk of new-onset diabetes in the post-acute state was higher in patients with more severe acute COVID-19 infection [10,13]. The role of vaccination in reducing the severity of acute COVID-19 illness and hospitalization has been demonstrated in multiple studies [31,32]. More studies are needed to understand the impact of vaccination on the risk of diabetes mellitus as a post-COVID-19 condition. 

Hayden et al. suggested multiple mechanisms for developing new-onset diabetes as a long-term complication of acute COVID-19 infections [33]. These include redox stress, inflammation, islet fibrosis, an active role of metabolic syndrome, systemic and tissue islet renin–angiotensin–aldosterone system, amyloid deposition, and β-cell dysfunction and apoptosis. Impaired insulin secretion due to the destruction of beta cells of the pancreas, hyperglycemia due to stress, and steroids in patients with COVID-19 infection can all contribute to the development of new-onset diabetes mellitus [34]. 

**Figure 1:**
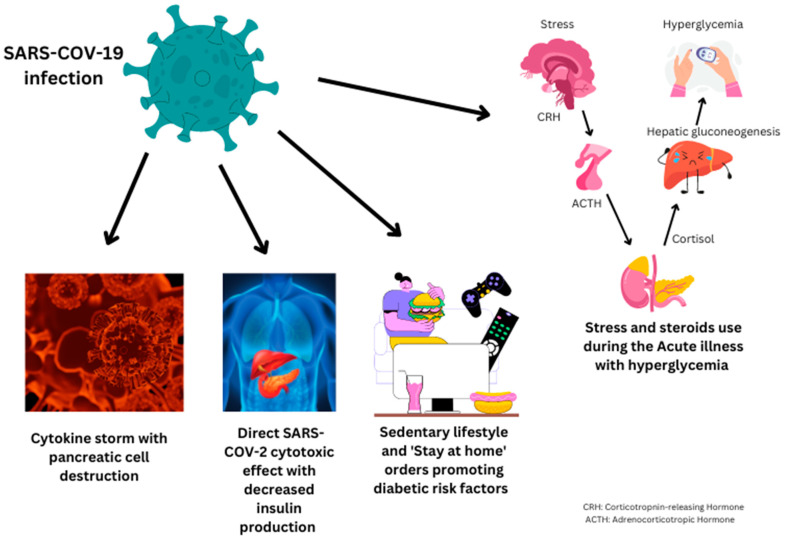
Possible mechanisms for COVID-19 infection and new-onset diabetes mellitus.

COVID-19 infection and new-onset diabetes mellitus: Possible Mechanisms (Figure 1):

A. Undiagnosed diabetes mellitus:

Patients admitted to hospital with an acute COVID-19 infection may have undiagnosed diabetes mellitus. Significant changes in lifestyle, such as poor diet, reduced exercise, and physical activity, associated with the COVID-19 pandemic, such as “Lock-down,” may have contributed to increased weight and glyco-metabolic syndrome in the prediabetes population and may increase the risk of new-onset diabetes in the post-infectious stage [29]. Xie et al. found an increased risk of new-onset diabetes in patients with COVID-19 infection despite using a control group from the same time period [8].

B. SARS-CoV-2 virus affecting the pancreas:

A viral infection such as the SARS-CoV-2 virus can affect the pancreas directly or indirectly. Many viruses, such as measles, mumps, cytomegalovirus, and the human immunodeficiency virus, can lead to acute inflammation in the pancreas with resultant pancreatitis [7]. Pancreatic islet cells contain multiple angiotensin-converting enzyme receptors. These receptors bind the SARS-CoV-2 virus and facilitate its entry into the cell, where it can cause direct cytotoxic effects and inflammation. The destruction of pancreatic beta cells can lead to hyperglycemia and diabetes mellitus [35]. 

Patients with an acute SARS-CoV-2 virus infection can be affected by cytokine storm, a highly inflammatory condition involving multiple organs in the human body, including the pancreas. It can lead to acute pancreatitis and degeneration of the islet cells of the pancreas [36]. This may result in hyperglycemia and diabetes if the damage is severe.

C. Hyperglycemia due to stress from acute COVID-19 infection:

COVID-19 disease or any other viral/bacterial infection can increase stress hormones such as adrenaline and cortisol [37]. These hormones can lead to an increase in the production of glucose levels with resultant hyperglycemia. Similarly, patients with COVID-19 are frequently prescribed steroids during the acute phase [8]. Steroids can cause an increase in the breakdown of lipids, proteins, and hepatic glucose production [38]. All three factors increase glucose production in the body, increasing the risk of hyperglycemia. Steroids are also responsible for the increase in insulin resistance, mainly by interfering with the signaling cascade of GLUT-4 transporters (glucose transporter type 4) within the muscle cells [38]. By interfering with the GLUT-4 transporter, steroids cause a 30–50% reduction in glucose uptake in the muscle cells from insulin, increasing insulin resistance and hyperglycemia with an increased risk of diabetes mellitus [39]. In healthy individuals, this can be compensated by increased insulin secretions, leading to normal serum glucose levels. However, in high-risk individuals, such as obese patients with low insulin sensitivity, steroids can lead to hyperglycemia and diabetes mellitus [40]. Steroids are commonly used in the first 1–2 weeks of acute infection. Our review has included studies with the onset of diabetes mellitus 4 weeks after the acute COVID-19 infection, minimizing the effect of steroids on a new diagnosis of diabetes mellitus.

However, some hypotheses that can be derived are: (1) Proinflammatory conditions from cytokine release can lead to pancreatic beta cell disruption and, thus, the onset of diabetes. (2) Stress-induced hyperglycemia with or without steroid use during the acute phase of illness can lead to hyperglycemia and diabetes during the acute phase of illness [37]. (3) Lifestyle changes with undiagnosed diabetes or metabolic syndrome culminate in this outcome [34]. (4) SARS-CoV-2 can cause direct cytotoxic injury after infecting the pancreatic cells, leading to decreased insulin production [35]. 

### 4.1. Limitations

In the majority of studies, ICD-10 codes were used to identify patients with diabetes and COVID-19, which can result in classification bias. We considered studies with new-onset diabetes four weeks after the initial diagnosis of COVID-19 infection. The COVID-19 pandemic all over the world was linked to movement restrictions or “stay home orders,” which can change how people eat and move and increase their risk of getting diabetes. Moreover, four weeks may not be sufficient to reflect changes in hemoglobin A1C levels. Despite these limitations, multiple studies, including one with a control population without COVID-19 infection during the same time period, demonstrated an increased risk of diabetes mellitus [8] four weeks after an acute COVID-19 infection.

### 4.2. Way Forward

Our scoping review suggests the need for a more comprehensive systematic review and meta-analysis to understand the increased risk of new-onset diabetes mellitus 4 weeks after an acute COVID-19 infection.

More evidence may highlight the need for screening for new-onset diabetes mellitus in patients with a history of COVID-19 and can be considered as a part of routine screening in post-COVID-19 clinics [3]. A multinational group of well-established diabetes researchers have created a global registry of patients with COVID-19-related diabetes mellitus [27]. This endeavor will assist in improving understanding of the increased risk of different diseases, especially diabetes and metabolic syndrome, during this COVID-19 pandemic.

## 5. Conclusions

Millions of people have been affected worldwide by the COVID-19 infection, with the SARS-CoV-2 virus continuing to evolve with the emergence of new variants. With its large population burden, post-infectious complications of COVID-19, including diabetes mellitus, can translate into a significant public health burden. Our scoping review synthesizes current supporting evidence regarding the relationship between COVID-19 infection and new-onset diabetes mellitus four weeks after acute infection.

More cohort studies and a meta-analysis of the available data are needed to increase the understanding of this relationship. It is also essential to develop effective strategies for the early identification and treatment of new-onset diabetes after acute COVID-19 infection to reduce its global impact.

## Figures and Tables

**Table 1:** Characteristics of included studies.

Serial Number	Year	Author ID	Country	Study Design	Patient Population	Follow-Up (in Days)	Sample Size	Mean Age/Gender (Female %)
1	2020	Ayoubkhani, D., et al. [20]	England	RCS with matched control	Post hospital discharge patients	140 (mean)	47,780 patients	64.5/45%
2	2021	Daugherty, S.E., et al. [12]	USA	RCS	3 databases: National administrative claims An outpatient laboratory testing An inpatient hospital admission	87 (median)	266,586 COVID-19 patients, 100% matched primary and secondary comparison groups	42.4/49.8%
3	2021	Maestre-Muñiz, M.M., et al. [18]	Spain	Cross-sectional study	Post hospital discharge patients	365 (mean)	543 patients	65.1/49.3%
4	2021	Rathmann, W., et al. [14]	Germany	RCS	Outpatients with non-exposed control group with AURI	119 (median)	35,865 patients in both groups	42.6/45.6%
5	2021	Estiri, H., et al. [11]	USA	RCS	Non-hospitalized patients	3–6 months and 6–9 months after COVID	96,025 patients	NA
6	2021	Montefusco, L., et al. [16]	Italy	Cohort study	Post hospital discharge patients	~60 (mean)	551 patients	61/38%
7	2022	Xie, Y., et al. [10]	USA	Cohort study	Inpatients including ICU patients and outpatients	352 (median)	COVID-19 patients—181,280 patients, contemporary control 4,118,441 patients, historical control 4,286,911 patients	60.92/11.9%
8	2022	Zhang, J., et al. [19]	China	Longitudinal prospective study	Post hospital discharge patients	~365 (mean)	248 patients	61 (median)/54.8%
9	2022	Rezel-Potts, E., et al. [15]	England	Cohort study	Outpatients	<4 weeks, 5–12 weeks, and 13 to 52 weeks from index date	428,650 patients in case and control group each	35/56%
10	2022	Laurenzi, A., et al. [17]	Italy	Prospective cohort study	Post hospital discharge patients	215 (median)	660 patients	64/33.3%
11	2022	Birabaharan, et al. [13]	USA and outside USA	RCS	Outpatients and inpatients. Control population included influenza patients	180 days	600,055 COVID-19 patients and 394,667 influenza patients	NA

RCS—retrospective cohort study, AURI—acute upper respiratory tract infection.

**Table 2:** Key findings in included studies.

Serial Number	Author ID	Comparison Groups	Key Findings
1	Ayoubkhani, D., et al. [20]	Patients with COVID-19 infection vs. patients with no COVID-19 infection	Diabetes was diagnosed after discharge in 4.9% of patients with COVID-19 with rate of 127 (122 to 132) diagnoses per 1000 person years. New-onset diabetes was diagnosed 29 (26 to 32) diagnoses per 1000 person years.
2	Daugherty, S.E., et al. [12]	COVID-19 vs. non-COVID-19 (cases vs. matched controls)—3 comparator groups: (1) 2019 comparator group (2) 2020 with adult patients with no diagnosis of COVID-19 (3) Viral lower respiratory tract illness comparator group	Hazard ratios for patients with COVID-19 infection and 2020 comparator group after the acute infection were highest in the first month from the index date and elevated for up to 6 months for diabetes mellitus (2.47, 95% CI 1.14–5.38). Diabetes mellitus was increased after COVID-19 infection with all comparison groups (2020, 2019, and viral lower respiratory tract illness group). Excess risk for new clinical sequelae after acute COVID-19 did not differ significantly between male and females.
3	Maestre-Muñiz, M.M., et al. [18]	No	Described incidence of DM2 in 1.3% (7) patients
4	Rathmann, W., et al. [14]	Patients with COVID-19 infection vs. acute upper respiratory tract infection as control	Increased incidence of type 2 diabetes mellitus in patients with COVID-19 as compared to AURI group (15.8 vs. 12.3 per 1000 person years). IRR for type 2 diabetes was 1.28 (95% CI 1.05–1.57). IRR was not increased for other forms of diabetes (IRR 1.17, 95% CI 0.80–1.71).
5	Estiri, H., et al. [11]	No	Increased risk of diabetes mellitus in both 3–6- and 6–9-month time windows (OR 1.41, 95% CI 1.22–1.64).
6	Montefusco, L., et al. [16]	Continuous glucose monitoring (CGM) was performed in COVID-19 or in patients recovering from COVID-19 with normal fasting glucose levels, in healthy controls and in patients with type 2 diabetes (during hospitalization)	46% of patients were hyperglycemic, whereas 27% were normoglycemic in post-discharge follow-up. In subgroup with CGM, COVID-19 was associated with impaired glycemic profile in normoglycemic patients, significantly higher glycemic area under the curve (AUC) above 140 m/dL, higher postprandial glycemic at 60 minutes, higher glycemic variability, higher standard deviation as compared to healthy controls.
7	Xie, Y., et al. [10]	COVID-19 patients with contemporary control and historical control	In post-acute phase, compared with contemporary control group, COVID-19 patients had increased risk (1.40, 95% CI 1.36–1.44) and excess burden (12.35, 11.36–13.38, per 1000 people at 12 months) of diabetes compared with contemporary control group, increased risk (1.85, (1.78–1.92)) and excess burden (12.35, 11.36–13.38) of incident anti-hyperglycemic use, increased risk of a composite endpoint of incident diabetes or anti hyperglycemic (HR of 1·46 (95% CI 1.43–1.50)) and excess burden of 18.03 (95% CI 16.59–19.51) per 1000 people at 12 months. Risk and burden of post-acute outcomes increased in a graded fashion based on severity of acute infection (non-hospitalized, hospitalized, admission to ICU).
8	Zhang, J., et al. [19]	Categorized into two groups according to whether they were critically ill or not during their initial hospital stay	Critical illness was associated with a higher risk of diabetes incidence one year after discharge as compared to mild/moderate risk COVID-19 infection. Association was more evident in males (OR = 5.70, 95% CI: 1.46, 22.15). Limitation: (1) No control group, OR was between severely ill vs. not severely ill. (2) Critical illness was defined by the following criteria: (1) respiratory failure requiring mechanical ventilation, (2) shock, (3) complications with other organ failures that required monitoring and treatment in an intensive care unit (ICU).
9	Rezel-Potts, E., et al. [15]	Patients with COVID-19 infection vs. patients without COVID-19 infection	Diabetes mellitus net incidence increased in the first 4 weeks after COVID-19 infection (relative risk 1.81, 95% CI 1.51 to 2.19). Diabetes mellitus net incidence continued to be elevated from weeks 5 to 12 after acute COVID-19 infection (relative risk 1.27, 95% CI 1.51–2.19). Diabetes mellitus net incidence was not increased from weeks 13 to 52 from acute COVID-19 infection (relative risk 10.07, 95% CI 0.99–1.16).
10	Laurenzi, A., et al. [17]	Patients with COVID-19 vs. patients without COVID-19 infection	A significant increase in FBG was noted during hospitalization (from 95 to 102 mg/d, *p* = 0.003) with return to pre-infection levels during follow-up (97.5 mg/dL *p* = 0.24 vs. pre-infection levels, *p* = 0.004 vs. hospitalization). After resolution of the infection, prevalence of dysglycemia reverted to preadmission frequency.
11	Birabaharan, et al. [13]	Patients with COVID-19 infection vs. patients with influenza	In patients with mild COVID-19 disease, estimated risk of new-onset type 2 diabetes mellitus within 180 days was 1.1%, estimated rate per 1000 person years was 23 and had relative risk 1.54 (95% CI 1.46–1.62) times higher than mild influenza controls. In patients with moderate/severe COVID-19 disease, estimated risk of new-onset type 2 diabetes mellitus within 180 days was 4.1%, estimated rate per 1000 person years was 83 and had relative risk 1.46 (95% CI 1.26–1.69) times higher than moderate/severe influenza controls. In subgroup analysis after excluding steroid use, risk of new-onset type 2 diabetes was less in patients with mild COVID-19 (RR 1.22, 95% CI 1.14–1.29) and remained same in patients with moderate/severe COVID-19(RR 1.42 (95% CI 1.13–1.80).

**Table 3:** Reported outcomes from included studies.

Serial Number	Author ID	Reported Events of Incident Diabetes Reported (COVID-19 vs. Control)	Adjusted HR/RR (95% CI) of Study Outcomes
1	Ayoubkhani, D., et al. [20]	28.7 (CI 26–31.7) vs. 8.2 (CI 6.9–9.8) per 1000 person years	1.50 (1.40, 1.61)
2	Daugherty, S.E., et al. [12]	Cumulative incidence 1.04 vs. 0.57 events per 100 person years	1.82 (1.69, 1.96)
3	Maestre-Muñiz, M.M., et al. [18]	NA	NA
4	Rathmann, W., et al. [14]	20.5 vs. 13.6 events per 1000 person years	1.26 (0.93, 1.71)
5	Estiri, H., et al. [11]	NA	NA
6	Montefusco, L., et al. [16]	65 patients vs. 147 patients with normal blood glucose levels	NA
7	Xie, Y., et al. [10]	48.38 (CI 47.04–49.76) vs. 34.9 (CI 34.7–35.1) events per 1000 person years at 12 months	1.40 (1.36, 1.44)
8	Zhang, J., et al. [19]	36 diabetic cases vs. 166 normal blood sugar level	3.53 (1.48–8.40)
9	Rezel-Potts, E., et al. [15]	681 vs. 384 cases of diabetes; 19.54 (95% CI: 18.10 to 21.06) vs. 11.10 (95% CI 10.01 to 12.26) per 100,000 patient weeks	1.27 (1.11 to 1.46)
10	Laurenzi, A., et al. [17]	154 diabetes vs. 176 normoglycemia	NA
11	Birabaharan, et al. [13]	83 vs. 56 events per 1000 person years 63 vs. 44 events per 1000 person years	1.46 (1.26, 1.69) 1.42 (1.13. 1.80

NA, not available.

## Data Availability

Restrictions apply to the availability of these data. Data were obtained from the National Inpatient Sample database, USA.

## Supplementary Materials

The following supporting information can be downloaded at: https://www.mdpi.com/article/10.3390/jcm12031159/s1, Figure S1: PRISMA Diagram; Table S1. PRISMA—ScR Checklist for current scoping review; Table S2. Search strategy.
