# Supplementary material for: Risk of New-Onset Diabetes Mellitus as a Post-COVID-19 Condition and Possible Mechanisms: A Scoping Review
Source: J Clin Med. 2023 Feb 1;12(3):1159. doi: 10.3390/jcm12031159 (PMC9917823; doi:10.3390/jcm12031159)
Supplement: Supplementary file 1 [file jcm-12-01159-s001.zip › jcm-2126046-supplementary.pdf]

Table S1. PRISMA—ScR Checklist for current scoping review.

Item	PRISMA-ScR CHECKLIST ITEM	Done?	Section
1	Identify the report as a scoping review.	Yes	Title
2	Provide a structured summary that includes (as applicable): background, objectives, eligibility criteria, sources of evidence, charting methods, results, and conclusions that relate to the review questions and objectives.	Yes	Abstract
3	Describe the rationale for the review in the context of what is already known. Explain why the review questions/objectives lend themselves to a scoping review approach.	Yes	Background
4	Provide an explicit statement of the questions and objectives being addressed with reference to their key elements (e.g., population or participants, concepts, and context) or other relevant key elements used to conceptualize the review questions and/or objectives.	Yes	Objective
5	Indicate whether a review protocol exists; state if and where it can be accessed (e.g., a Web address); and if available, provide registration information, including the registration number.	Yes	Methods
6	Specify characteristics of the sources of evidence used as eligibility criteria (e.g., years considered, language, and publication status), and provide a rationale.	Yes	Methods
7	Describe all information sources in the search (e.g., databases with dates of coverage and contact with authors to identify additional sources), as well as the date the most recent search was executed.	Yes	Methods
8	Present the full electronic search strategy for at least 1 database, including any limits used, such that it could be repeated.	Yes	Supplementary Table S2
9	State the process for selecting sources of evidence (i.e., screening and eligibility) included in the scoping review.	Yes	Methods
10	Describe the methods of charting data from the included sources of evidence (e.g., calibrated forms or forms that have been tested by the team before their use, and whether data charting was done independently or in duplicate) and any processes for obtaining and confirming data from investigators.	Yes	Methods
11	List and define all variables for which data were sought and any assumptions and simplifications made.	Yes	Methods
12	If done, provide a rationale for conducting a critical appraisal of included sources of evidence; describe the methods used and how this information was used in any data synthesis (if appropriate).	No; No critical appraisal conducted	

13	Describe the methods of handling and summarizing the data that were charted.	Yes	Methods
14	Give numbers of sources of evidence screened, assessed for eligibility, and included in the review, with reasons for exclusions at each stage, ideally using a flow diagram.	Yes	Figure 1: PRISMA diagram
15	For each source of evidence, present characteristics for which data were charted and provide the citations.	Yes	Characteristics of included studies Table 1
16	If done, present data on critical appraisal of included sources of evidence (see item 12).	No; No critical appraisal conducted	
17	For each included source of evidence, present the relevant data that were charted that relate to the review questions and objectives.	Yes	Summary of findings table 2 and 3
18	Summarize and/or present the charting results as they relate to the review questions and objectives.	Yes	Results
19	Summarize the main results (including an overview of concepts, themes, and types of evidence available), link to the review questions and objectives, and consider the relevance to key groups.	Yes	Results
20	Discuss the limitations of the scoping review process.	Yes	Discussion/Limitation
21	Provide a general interpretation of the results with respect to the review questions and objectives, as well as potential implications and/or next steps.	Yes	Discussion
22	Describe sources of funding for the included sources of evidence, as well as sources of funding for the scoping review. Describe the role of the funders of the scoping review.	Yes	Funding Acknowledgements

Table S2. Search strategy.

MEDLINE-PubMed		
#1	285,813	COVID-19 OR "COVID 19" [MeSH Terms] OR COVID-2019 OR SARS-CoV-2 OR 2019-nCoV OR 2019-SARS-CoV-2
#2	70,339	"long-term Covid" OR long-term OR consequence* OR "long-term impact" OR "long-term effect" OR "post-acute" OR long-tail OR persist* OR "chronic-COVID" OR "long-COVID" OR "long-haul" .ab OR "Long COVID" or "long haulers" or "post-acute COVID" or "persistent COVID" or "chronic COVID syndrome"
#3	2827	#1 AND #2
#4	844,233	"Diabetes Mellitus"[Mesh] OR "Diabetes Mellitus, Type 2"[Mesh] OR "Diabetes Mellitus, Type 1"[Mesh] OR "glucose intolerance"[Mesh] OR "insulin resistance"[Mesh] OR "Hyperglycemia"[Mesh] OR "Diabetes Mellitus"[Text] OR "Diabetes Mellitus, Type 2"[Text] OR "diabetes type 1"[Text] OR "T2DM"[Text] OR "diabetes Type II"[Text] OR "diabetes Type I"[Text] OR "diabetes"[Text] OR

		"glucose intolerance"[Text] OR "insulin resistance"[Text] OR "Hyperglycemia"[Text]
#6	115	(#3) AND (#4)
	**Embase**	
#1	293,857	'2019 ncov' OR 2019ncov OR 'covid 19' OR 'sars cov 2' OR (wuhan AND coronavirus AND [12-1-2019]/sd)
#2	46,677	((survivor* OR recover* OR persistent OR follow) AND up OR discharge* OR sequela* OR long) AND ('covid'/exp OR covid) AND [embase]/lim
#3	43,513	#1 AND #2
#4	1,302,407	('diabetes mellitus')/br OR (('diabetes mellitus'):ti,ab,kw) OR (('non insulin dependent diabetes mellitus'):ti,ab,kw) OR (('insulin dependent diabetes mellitus'):ti,ab,kw) OR ((hyperglycemia):ti,ab,kw) OR (('insulin resistance'):ti,ab,kw)
#5	266	(#3) AND (#4)
	**Scopus**	
#1	48,627	(TITLE-ABS-KEY (long AND covid) OR TITLE-ABS-KEY (post AND covid) OR TITLE-ABS-KEY (post-acute AND covid-19))
#2	261,907	(TITLE-ABS-KEY (diabete!) OR TITLE-ABS-KEY (hyperglycemia) OR TITLE-ABS-KEY ("insulin resistance"))
#3	196	(#1) AND (#2)

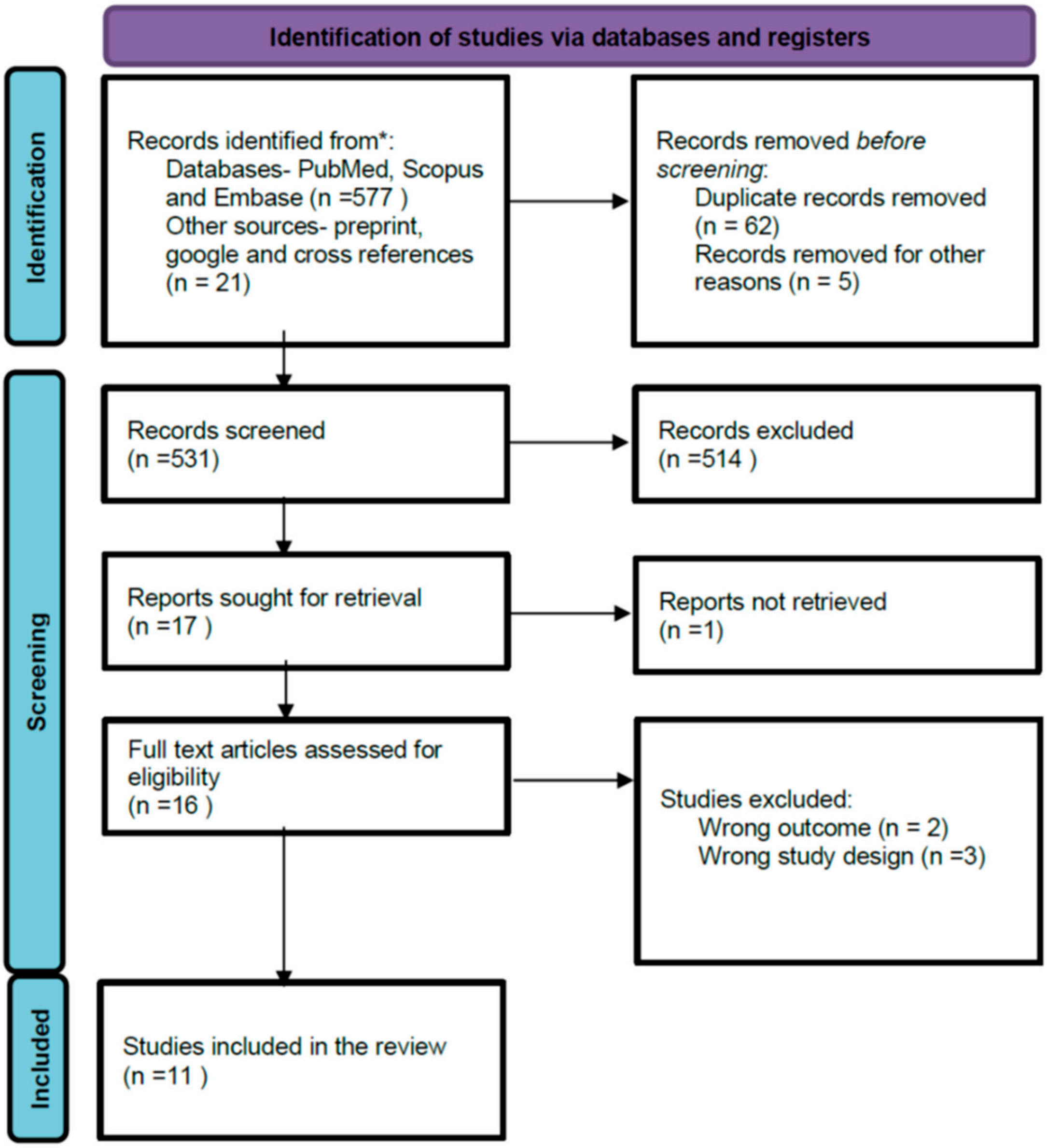

Figure S1. PRISMA Diagram.
